# Oxidative Stress, Mitochondrial Function and Adaptation to Exercise: New Perspectives in Nutrition

**DOI:** 10.3390/life11111269

**Published:** 2021-11-22

**Authors:** Nancy Vargas-Mendoza, Marcelo Angeles-Valencia, Ángel Morales-González, Eduardo Osiris Madrigal-Santillán, Mauricio Morales-Martínez, Eduardo Madrigal-Bujaidar, Isela Álvarez-González, José Gutiérrez-Salinas, César Esquivel-Chirino, Germán Chamorro-Cevallos, José Melesio Cristóbal-Luna, José A. Morales-González

**Affiliations:** 1 Laboratorio de Medicina de Conservación, Escuela Superior de Medicina, Instituto Politécnico Nacional, Plan de San Luis y Díaz Mirón, Col. Casco de Santo Tomás, Del. Miguel Hidalgo, Ciudad de México 11340, Mexico; nvargas_mendoza@hotmail.com (N.V.-M.); angeles_v_marcelo@hotmail.com (M.A.-V.); eomsmx@yahoo.com.mx (E.O.M.-S.); 2 Escuela Superior de Cómputo, Instituto Politécnico Nacional, Av. Juan de Dios Bátiz s/n Esquina Miguel Othón de Mendizabal, Unidad Profesional Adolfo López Mateos, Ciudad de México 07738, Mexico; 3 Licenciatura en Nutrición, Universidad Intercontinental, Insurgentes Sur 4303, Santa Úrsula Xitla, Alcaldía Tlalpan, Ciudad de México 14420, Mexico; mtz98mauxd@gmail.com; 4 Laboratorio de Genética, Escuela Nacional de Ciencias Biológicas, Instituto Politécnico Nacional, Unidad Profesional A. López Mateos, Av. Wilfrido Massieu, Col., Lindavista, Ciudad de México 07738, Mexico; edumadrigal.bujaidar@gmail.com (E.M.-B.); isela.alvarez@gmail.com (I.Á.-G.); 5 Laboratorio de Bioquímica y Medicina Experimental, Centro Médico Nacional “20 de Noviembre”, ISSSTE, Ciudad de México 03229, Mexico; quauhtlicutli@yahoo.com; 6 Área de Básicas Médicas, División de Estudios Profesionales, Facultad de Odontología, Universidad Nacional Autónoma de México, Ciudad de México 04510, Mexico; investigaciondental@gmail.com; 7 Laboratorio de Toxicología Preclínica, Departamento de Farmacia, Escuela Nacional de Ciencias Biológicas, Instituto Politécnico Nacional, Av. Wilfrido Massieu 399, Col. Nueva Industrial Vallejo, Del. Gustavo A. Madero, Ciudad de México 07738, Mexico; gchamcev@yahoo.com.mx (G.C.-C.); josmcl@hotmail.com (J.M.C.-L.)

**Keywords:** oxidative stress, ROS/RNS, exercise, mitochondrial adaptation, oxidative damage, antioxidants, exercise performance

## Abstract

Cells have the ability to adapt to stressful environments as a part of their evolution. Physical exercise induces an increase of a demand for energy that must be met by mitochondria as the main (ATP) provider. However, this process leads to the increase of free radicals and the so-called reactive oxygen species (ROS), which are necessary for the maintenance of cell signaling and homeostasis. In addition, mitochondrial biogenesis is influenced by exercise in continuous crosstalk between the mitochondria and the nuclear genome. Excessive workloads may induce severe mitochondrial stress, resulting in oxidative damage. In this regard, the objective of this work was to provide a general overview of the molecular mechanisms involved in mitochondrial adaptation during exercise and to understand if some nutrients such as antioxidants may be implicated in blunt adaptation and/or an impact on the performance of exercise by different means.

## 1. Introduction

The high-oxygen atmospheric concentrations of a million years ago induced metabolic changes, resulting in the majority of living organisms utilizing more oxygen (O_2_), thus promoting the development of aerobic systems. The basis of aerobic metabolism is supported by the biotransformation of molecular O_2_ to meet energy demands, homeostasis and to ensure the support of life. In turn, highly reactive molecules result from these cellular processes, such as the reactive oxygen species (ROS), which are considered radical and non-radical derivatives of O_2_. ROS are formed from one-electron transfers from a redox donor to molecular O_2_. Further, the term reactive nitrogen species (RNS) refers to reactive molecules derived from nitrogen. Free radicals (FR) are considered an atom/molecule that contains one or more unpaired electrons, which renders them unstable and very reactive [1].

The anion superoxide (O_2_^•−^) is the main ROS formed in cells by the one-electron reduction of molecular O_2_; it is negatively charged and impermeable to the cell membrane as compared with other FR and has a relatively long half-life. The dismutation of O_2_^•−^ leads to the formation of hydrogen peroxide (H_2_O_2_), a permeable non-FR cell membrane, relatively stable, weak in oxidation, and possessing the ability to diffuse within and outside of the cell [2]. Because H_2_O_2_ has a long half-life, it can reach high cellular concentrations, inducing cytotoxicity and cell signaling. H_2_O_2,_ together with O_2_^•−^ are transformed into hydroxyl radicals (^•^OH) that are highly reactive molecules with powerful oxidizing potential, but that are not membrane-permeable; thus, they directly react with molecules in close proximity to their production site, causing structural cell damage [1,2].

In addition, among RNS, the most important free radical is oxide nitric (NO), which is synthesized from the amino acid L-arginine by nitric oxide synthase (NOS). NO can also react with O_2_^•−^ to form peroxynitrite (ONOO^−^), which is the main cause of the nitration of cellular proteins and the depletion of thiol groups due to its rapid formation. All of these previously mentioned reactive molecules might contribute to oxidative stress (OS), defined as “an imbalance between oxidants and antioxidants in favor of the oxidants, leading to a disruption of redox signaling and control, and/or molecular damage” [3]. Fortunately, cellular systems have developed potent cytoprotector systems as a defense against damage caused by the high levels of ROS and RNS. For instance, endogenous antioxidant systems (EnAS) are activated through the nuclear factor erythroid 2-related factor 2 (Nrf2), a redox-sensitive transcriptional factor that coordinates the antioxidant response and that is also related to mitochondrial biogenesis [4].

Despite the imbalance that ROS and RNS contribute to oxidative stress, it is known that certain levels of these molecules are needed to trigger cell signaling, adaptation, and homeostasis. During exercise, physical work increases energy demands, the O_2_ uptake, and consequently the formation of ROS [5]. In this manner, mitochondria are susceptible to being attacked in high intense or long sports, which might represent a risk for the detriment in muscle force, contraction, and thus performance. Therefore, the purpose of this review was to provide a general overview of mitochondrial function and the molecular mechanisms implicated in adaptation to stress induced by exercise. Moreover, nutritional insights in terms of the growing evidence with regard to the use of antioxidants and mitochondrial protection are discussed.

## 2. Oxidative Stress in Exercise

During exertion, muscles are the main source of ROS production within the active muscle fibers from different sites such as the mitochondria, T-tubules, sarcoplasmic reticulum, and sarcolemma. Among these, mitochondria are thought to be the major ROS producer, predominantly in endurance training. Once exercise starts, energy demands rise 10–20 fold in oxygen consumption above that of the resting state [2]. Thus, the augmented oxidative phosphorylation increases O_2_^•−^ in contracting muscle fibers. However, some recent evidence indicates that mitochondria may not be fully responsible for the abundance of ROS because it is possible to produce more O_2_^•−^ during basal state-4 respiration compared with active stage-3 respiration [6].

There are other ROS producers, which include nicotinamide, adenine dinucleotide phosphate oxidase (NADPH oxidase), phospholipase A_2_, (PLA_2_), xanthine oxidase monoamine oxidases (MAO’s), some dehydrogenases, and some immune-system cells, such as macrophages, eosinophils, monocytes, and neutrophils [2]. For instance, the NADPH oxidase isoform NOX2, located within T-tubes and sarcolemma, is the primary ROS producer in contracting muscle fibers, whereas the other isoform, NOX4, localized in mitochondria and the sarcoplasmic reticulum, contributes to ROS synthesis under baseline conditions [7]. At the same time, PLA_2_ can activate NADPH oxidases, the calcium-dependent isoform promotes the release of ROS during muscle contraction [8], and the independent-isoform manages oxidation under resting conditions [9]. PLA_2_ is involved in ROS generation during muscle contraction when the calcium uptake in mitochondria is increased; then, PLA_2_ is activated within the mitochondria, promoting the interaction of arachidonic acid synthesis with the electron transport chain (ETC). The formation of arachidonic acid elicits an eicosanoid substrate that could also modulate ROS production. In fact, the arachidonic acid released by PLA_2_ is a substrate of lipoxygenases, a ROS-generating enzyme system deriving from membrane phospholipids [9].

### 2.1. Impact of Oxidative Stress on Muscle Contraction

The impairment of modulating exercise-induced OS is implicated in overtraining syndrome (OTS), which is characterized by extended periods of fatigue-induced from intense training/competitions and inappropriate recovery post-exercise/competition episodes. In consequence, excessive ROS and OTS are closely involved in performance weakening [10]. Skeletal-muscle fatigue is considered as a decline of maximal force production in response to contractile activity [11]. The production of skeletal-muscle force depends on the contractile mechanism and metabolic factors, including lactate, inorganic phosphate (Pi), hydrogen (H^+^) ions, ROS, and heat shock protein (HSP), among others, that can enhance muscle fatigue [11].

Some evidence supports the hypothesis that increased fatigue and force depression are the result of the oxidizing state of muscle fibers due to continued exposure to ROS, basically to H_2_O_2_. Contrariwise, it has been identified that unfatigued muscle remains predominantly reduced. Also, the sustained intramuscular imbalance of the redox state enhanced chronic inflammation, exerting an impact upon force production and athletic performance [12]. Indeed, the endogenous productions of ROS/RNS play a crucial role in submaximal force, due to that the continuous state of reduced maximal force is induced by Ca^2+^ myofibrillar sensitivity and/or the decrease of reticulum sarcoplasmic Ca^2+^ release; these processes are ROS/RNS-dependent. Meanwhile, in rested myofibers, the acute and mild exposure of ROS/RNS raises submaximal force, but extended exposure impacts negatively [13]. Other ROS/RNS-mediated mechanisms associated with skeletal-muscle fatigue include a decrease in cellular-membrane excitability, impairment of the interaction between voltage sensors and Ca^2+^ ion channels, due to the excitation/contraction-coupling failure and the blocking of the action potential. Furthermore, Ca^2+^ release interferes in actin-myosin cross-bridge formation and in the reduction of myofibrillar sensitivity [14]. Additionally, there is evidence that ROS also exert a modulatory effect on Na^+^/K^+^ pump activity, but that excessive ROS production could impair Na^+^/K^+^ pump activity and more proteins implied in excitation-contraction-coupling, affecting muscle force during prolonged endurance exercise [6].

The underlying beneficial or deleterious effects of ROS/RNS on muscle contraction are demonstrated in several studies. Different factors, such as location, time of exposure, type of ROS/RNS, and the combination of endogenous/exogenous antioxidant systems, will determine effective skeletal-muscle function.

### 2.2. Mitochondrial ROS Production and Antioxidant Defense

Under normal conditions, electron flow along the ETC to complex IV results in their final accumulation in molecular oxygen to produce H_2_O. Through exercise, predominantly in prolonged endurance activities, ATP production requires reduced equivalents of NADH and FADH2, free ADP, Pi, and O_2_, and the presence of these elements activates the ETC in mitochondria [15]. Along the ATP synthesis process, mitochondrial ROS (mtROS) is produced for the reaction of electrons with O_2_ at different sites to form superoxide/hydrogen peroxide fundamentally in complex I and in complex III of ETC, which can leak 1–3% of electron flux as O_2_^•−^ in state 4 or in the resting state [16,17]. Some evidence indicates that complex II and other mitochondrial enzymes also participate in mtROS formation [18]. It has been found that around 11 different mitochondrial sites leak electrons to O_2_ to produce the superoxide or hydroperoxide associated with oxidative phosphorylation (OxPho) and substrate oxidation. These sites are complexes I and III, oxoacid dehydrogenase complexes, and complex II, which use ubiquinone as an acceptor. Mitochondrial glycerol 3-phosphate dehydrogenase and complex III produce O_2_^•−^ to the matrix and the external side of the mitochondrial inner membrane, while the other complexes generate O_2_^•−^ and H_2_O_2_ only in the matrix [19]. Approximately 0.1–0.2% of the O_2_ consumed by mitochondria is transformed into O_2_^•−^. The release of O_2_^•−^ from mitochondria is in the form of H_2_O_2_ or OONO^−^ when superoxide anions react with ^•^NO; this is because the high proton environment in the internal mitochondrial membrane makes it difficult for O_2_^•−^ to cross the latter. Mitochondria are being found to be major ROS producers; however, inactive contracting muscles, a better-coupled electron flow is observed, and there is minor production of superoxide anions in the ETC. It is noteworthy that unfatigued and healthy muscle fibers could produce less ROS [20,21].

With the purpose of modulating the redox signaling of ROS/RNS, there is a complex defense system constituted of internal and external elements known as the endogenous antioxidant system (EnAS). This system is constituted of mean of enzymatic and non-enzymatic constituents and the exogenous antioxidant system (ExAS), which derives from dietary or supplementary sources such as antioxidant vitamins (vitamins C, E, and A), oligoelements (selenium, copper, and zinc), nucleic acids, and phytochemicals, among others. The latter will be discussed further ahead in this review. The EnAS acts to maintain reactive species within the homeostatic range in order to enhance cell signaling and prevent oxidative damage. Nevertheless, the overproduction of ROS/RNS may surpass the antioxidant defense, generating OS and damage. The dismutation of O_2_^•−^ is performed by a family of superoxide dismutases (SOD) to produce H_2_O_2_ and O_2_. There are three isoforms as follows: SOD1, located in the cytosol and mitochondrial intermembrane space; SOD2, found in the mitochondrial matrix, and SOD3, situated outside the cell. SOD1 requires copper and zinc as cofactors (Cu/ZnSOD), whereas SOD2 is manganese-dependent (MnSOD) [22]. SOD is highly active in type-I oxidative fibers compared with type-IIX fibers with less oxidative activity [23,24].

H_2_O_2_ and peroxides are removed by enzymatic and non-enzymatic elements of the EnAS, such the family of glutathione peroxidases (GPX) in the presence of reduced glutathione (GSH), glutathione reductase (GR), catalases (CAT), thioredoxins (Trx), peroxiredoxins (Prx), and glutaredoxins (Grx) [25]. There are eight isoforms of GPX in mammals (GPX1–GPX8) [26]; all of these reduce H_2_O_2_ to form H_2_O and alcohol, but the different isoforms are related to cell location. GPX1 is active in cytosol and mitochondria is selenium-dependent, GPX2 is exclusively located in cytosol, GPX3 is active in cytosol and the extracellular space, and GPX4 resides in the phospholipid environment to remove lipid peroxides [26]. GPX5 is expressed in mammalian epididymis and protects sperm from ROS and lipid peroxidation damage [27]. GPX6 has been found in epithelial cells [28]. GPX7 and GPX8 are similar in structure, but GPX7 works as a stress signal sensor and transmitter by shuttling disulfide bones to its interacting proteins, which are implicated in diverse signaling pathways to keep redox homeostasis [29]. GPX8 isoform is essentially located in the endoplasmic reticulum, is considered as a primary regulator of tumor aggressiveness in several types of cancer [30]. The reducing power of GPX is found in the use of GSH as electron donor. At the same time, this reaction produces glutathione disulfide (GSSH), the oxidated form of glutathione, which can be reduced by glutathione reductases (GR). In mammalian skeletal muscle and cardiac tissue, the resynthesis of GSH through GR needs NADPH produced by the NADPH-specific isocitrate dehydrogenase and by glyceraldehyde-3-phosphate dehydrogenase [25].

Catalase (CAT), which also participates in the removal of H_2_O_2_ to form H_2_O and O_2,_ is located in different compartments in the cell, basically in cytosol, peroxisomes and mitochondria [31] and, similar to SOD, it is more active in type-I than in type-IIX oxidative fibers. In contrast to GPX, CAT does not require an electron donor, nor does it eliminate other hydroperoxides [2]. Thioredoxins (Trx) and thioredoxin reductases comprise a group of antioxidant enzymes that maintain proteins in a reduced state by the formation of a disulfide bond with substrate proteins and they are able to transfer two of the electrons that they possess to the target protein. Consequently, Trx is oxidated, but then it can be reduced by Trx reductase in the presence of NADPH as an electron donor. Trx is the main disulfide reductase within cells and there are two isoforms: Trx1, located in the cytosol, and the Trx2 site in mitochondria [32,33]. These isoforms also participate in the removal of hydroperoxides, cell signaling, and vitamin-C recycling [34]. Peroxiredoxins (Prx) are enzymes involved in the reduction of H_2_O_2_, alkyl peroxides, and peroxynitrite using electrons of Trx. Six isoforms have been identified: Prx1, 2, and 4 are situated in cytosol, Prx3 is located in mitochondria, Prx5 is found in both mitochondria and cytosol, and Prx6 is localized in the extracellular space. These isoforms are also thought to be involved in cell signaling and circadian-cycle regulation [35,36]. Finally, glutaredoxins (Grx) are small-thiodisulfide oxidoreductases that protect protein and non-protein thiols through the transfer of electrons to the disulfide substrates deriving from NADPH. Grx participates in hydroperoxide elimination, and there are three isoforms: Grx1 is within the cytosol and Grx2 and 3 is situated in the mitochondria [37].

## 3. Mitochondrial Function and Adaptation

### 3.1. Mitochondrial Adaptation to Exercise in the Presence of ROS

The practice of exercise is a powerful stimulus that leads to metabolic and physiological adaptations and that eventually allows the body to tolerate heavier workloads, inducing changes in muscle-fiber remodeling and plasticity, which are the result of a complex relationship or diverse regulator factors and signaling processes [38]. During the last two decades, numerous groups of researchers have elucidated a vast list of signals triggered by ROS within muscle contraction and their implications in molecular events related to physiological responses and subsequent cell adaptation. Currently, among the main adaptations mediated by ROS are found the following: mitochondrial biogenesis, increased antioxidant defenses, and myofiber hypertrophy. The role that ROS plays in the previously mentioned adaptations is based on the idea that they affect protein function, enzyme activity, and gene transcription as well they modify membrane and genome integrity [39].

With regard to mitochondrial adaptations, the protagonism must be mentioned of peroxisome proliferator-activated receptor gamma coactivator-1 alpha (PGC-1α), a transcriptional cofactor that interacts with other transcriptional factors and that owns the ability to induce transcription without binding directly with DNA. This binding to specific transcriptional factors promotes the genomic expression of genes associated with mitochondrial biogenesis, energy metabolism, thermogenesis, and the regulation of uncoupling [40]. PGC-1α coordinates different redox-sensitive programs; for instance, PGC-1α interacts with the estrogen-related receptor alpha (ERRα), Nrf1, and Nrf2 in mitochondrial biogenesis [41]. Thus, PGC-1α is a key element for enhancing adaptative responses in endurance activities [42]. The increase of ROS, particularly H_2_O_2_ during prolonged aerobic resistance sports, exacts the activation of PGC-1α and the alternative promoter PGC-1α2 in muscle fibers via the regulation of the upstream stimulatory factor 1 (USF-1), which also depends on beta adrenergic signaling, in turn, activates the angiogenic vascular endothelial growth factor (VEGF), also mediating the orphan nuclear receptor ERRα. Moreover, the adrenergic stimulation of exercise-induced PGC-1α/ERRα/VEGF promotes angiogenesis [43]. PGC-1α is also involved in type-IIX myosin-fiber heavy chains when it interacts with the myocyte enhancer factor-2 (MEF2) [44].

Indeed, the practice of exercise has a potent effect on the activation of the EnAS that, to a great extent, is regulated by the nuclear factor erythroid 2-related factor (Nrf2), a nuclear transcription factor that coordinates more than 250 genes associated with the cytoprotective and antioxidant response. Under homeostatic conditions, Nrf2 is attached to its negative regulator Keap1, a redox-regulator substrate adaptor for the Cullin (Cul)3-RING-box protein (Rbx)1 ubiquitin ligase, which directs Nrf2 toward its degradation by ubiquitination [45]. However, when the cellular concentration of ROS rises, Keap1 cysteine-rich proteins are oxidized, inducing conformational changes. Subsequently, Nrf2 is released from Keap1 to translocate into the nucleus, where it has the ability to heterodimerize with musculoaponeurotic fibrosarcoma proteins (MAF proteins), binding to the antioxidant response element (ARE), a specific DNA sequence (ARE, 50-TGACNNNGC-30); that is the latter is responsible for the activation of a wide-ranging group of genes associated with the antioxidant and protective response, such as the enzymes involves in the biosynthesis and metabolism of glutathione, Trx, phase-II detoxifying enzymes, and NADPH-producing enzymes [46,47]. Nrf2 interacts with PGC-1α in a close relationship to mediate the antioxidant response, together with other signaling pathways. In addition, Nrf2 is involved in the regulation of mitochondrial enzymes that are associated with the activation of PGC-1α. In mitochondrial biogenesis, Nrf2 interacts with PGC-1α and other transcriptional factors such the mitochondrial transcription factor A (TFAM), which encodes active enzymes of the mitochondrial matrix and also stabilizes mtDNA via the synthesis of nucleoids and controls the amount of mtDNA, which is correlated with the amount of TFAM but not necessarily with the transcription level [48]. Nrf2 promotes mitochondrial biogenesis by maintaining levels of nuclear respiratory factor 1 (Nrf1) and promoting purine nucleotide biosynthesis [4]. The mitophagic process is also influenced in the presence of Nrf2: dysfunctional mitochondria are engulfed by autophagosomes that are prepared for their degradation and recycling in lysosomes, preserving mitochondrial homeostasis [4].

Autophagy as a physiological regulator helps to maintain the balance between organelle biogenesis, protein synthesis and degradation. Autophagy preserves cellular homeostasis where damaged organelles and biomolecules are engulfed to be subsequently degraded upon fusion with lysosomes. It can be activated as a pro-survival response to stress; it has been found that autophagy is a redox-sensitive process and ROS/RNS elicits the crosstalk between cell signaling and protein damage [49]. Mitophagy is known as a form of selective autophagy regard to mitochondria turnover is greatly induced by oxidative stress to remove dysfunctional mitochondria [50]. The relation between mitophagy and the complex Nrf2/Keap1 has been elucidated; one way described is via the transcriptional upregulation of p62/sequestosome-1 (p62/SQSTM1) involved in Keap1 autophagic degradation and the activation of Nrf2 promoting the antioxidant response [51]. Recently, Yamada et al. [52] reported that p62/SQSTM1 translocates Keap1 to the mitochondria for the ubiquitination and degradation promoting mitophagy. Another form of the relationship between Nrf2 and mitophagy is through Keap1 inhibitors such as phosphatase and tensin homologue (PTEN)-induced putative kinase 1 (PINK1), which is one of the main regulators of mitophagy [53].

Although the mechanisms underlying exercise-induced mitophagy have not been fully understood, it is being established that mitophagy initiates after 6 h of acute treadmill running in mice observed by increased phosphorylation of AMP-activated protein kinase (Ampk) at tyrosine 172 and of unc-51 like autophagy activating kinase 1 (Ulk1) at serine 555. The mentioned research highlights the effect of exercise on mitochondrial biogenesis and mitochondrial removal, driving mitophagy thru Ampk-Ulk1 signaling in skeletal muscle [54]. In rat myocardium, the elevation of OS after acute heavy exercise triggers inflammatory response via NLRP3 inflammasome activation, promoting mitophagy to compensate for counteracting myocardial damage [55]. The underlying effect of exercise on the activation of EnAS counteracts the elevated level of ROS and their potential effect on destabilizing membrane lipids, structural proteins, or DNA. Abundant evidence indicates that exercise effectively activates Nrf2, the antioxidant response, and mitochondrial biogenesis. It should be noted that an optimal antioxidant response depends largely on the constant feedback between the endogenous and exogenous defense systems (EnAS/ExAS), so the lack of antioxidant dietary intake may directly affect the effectiveness of the cytoprotective response.

### 3.2. Mitohormesis and Exercise

In 2006, Ji et al. [56] defined hormesis as the beneficial effect conferred by a low dose of an agent or phenomenon that would cause injury or toxicity at high levels. Exercise induces a certain level of stress depending on the intensity, duration, and frequency of the exercise that is necessary for adaptation to physical work: exercise-induced ROS elevation is required for adaptation at physiological levels to permit energy metabolism, stress regulation, protein turnover, immunomodulation, muscle growth, and the regulation of blood flow [57]. Hence, exercise preconditioning/hormesis can be conceived of as the mild OS necessary to increase protection against mechanical/metabolic stressors, extreme temperatures (heat/cold), hypoxic conditions, ischemia, and other factors. In skeletal muscle, exercise preconditioning could be the result of the synergy exerted by different elements, including transcriptional factors, antioxidant defense, the expression of heat shock proteins (HSP), and ROS-producing sources [58].

Nuclear factor (NF) kappaΒ and mitogen-activated protein kinases (MAPK) comprise one of the most important redox-sensitive signal pathways. Transcription factor NF-κΒ remains inactive when is conjugated thru inhibitory I-κΒ proteins, within the cytoplasm are activated by different stimulants such as proinflammatory cytokines including TNF-α, IL-1, and IL-6, lipopolysaccharides (LPS), and toxins, and the presence of H_2_O_2_ activates the signal. Then, NF-κB dissociates from I-κΒ by the activation of NFκB-inducing kinase (NIK) phosphorylating serine residues of I-κΒα (ser-32 and ser-36) or I-κΒβ (ser-19 and ser-23), releasing p50/p65 subunits. After this, it translocates into the nucleus and binds to the specific target genes involved in a wide variety of biological functions, such as the antioxidant and the inflammatory response, immunity, apoptosis, and adaptation [59,60]. The target genes are SOD2, vascular cell adhesion molecule-1 (VCAM-1), cyclooxygenase-2 (COX-2), inducible NOS (iNOS), and GCS [61]. On the other hand, MAPK signaling is implicated in transcription, translation, growth, and cell remodeling. MAPK is activated by growth factors, the inflammatory cytokines TNF-α and IL-1, LPS phorbol esters, and ROS [62,63]. The MAPK signal pathway integrates the extracellular signal-regulated kinase (ERK), the c-Jun amino-terminal kinase (JNK), and the p38MAPK, which are controlled by their respective upstream kinases (MEK/MKK) [62,64]. Inactive skeletal muscle, ROS play a role as signal messengers that trigger adaptative responses via the interrelationship of NF-κΒ and MAPK pathways, boosting exercise preconditioning [56].

**Figure 1:**
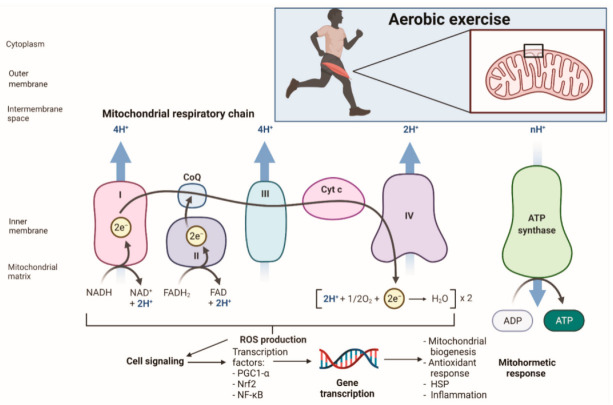
Exercise-induced oxidative stress and the development of the mithormetic response. Created with BioRender.com (accessed on 10 November 2021). NADH: reduced nicotinamide adenine dinucleotide; NAD: nicotinamide adenine dinucleotide; FADH: reduced flavin adenine dinucleotide; FAD: flavin adenine dinucleotide; CoQ: coenzyme Q; Cyt C: cytochrome C: PGC1-α: peroxisome proliferator-activated receptor gamma coactivator-1 alpha; Nrf2: nuclear factor erythroid 2-related factor; NF-κB: nuclear factor-kappa beta.

With respect to the mitochondria, the term mitohormesis refers to exposure to a sublethal dose of stress that could enhance mitochondrial function and that contribute to adaptative hormetic response [65]. In aerobic exercise OS, mechanical, unfolded/misfolded protein, and energy stress can induce several mitohormetic effects, such as antioxidant enzymes expression, myofibrillar protein synthesis, mitochondrial biogenesis, and protein folding (Figure 1). The result in terms of functionality is the increase of muscle mass and strength, improvement in mitochondrial function, oxidative capacity, and antioxidant response, a reduction in ROS production, and the enhancement of proteostasis [66]. Mitohormesis results from the interaction of several transcriptional factors, such as PPARα, EERα, Sp1, PGC-1α, PGC-1β, and Nrf2. In a study with 3- and 12-month-old Nrf2 Wild-Type (WT) and Nrf2 Knock Out (KO) mice submitted to a training protocol of voluntary wheel running, it was demonstrated that Nrf2/KO mice experienced failure in mitochondrial respiration of the intramyocellular fraction (IMC) in 40% of skeletal muscle and that the emission of ROS increased significantly. The aerobic resistance capacity of Nrf2/KO was reduced, along with the highest degree of fatigue associated with the elevated ROS found in mitochondrial samples, which probably affected mitochondrial integrity. Additionally, no significance in mitochondrial content was found; however, the activity of Cytochrome C Oxidase (COX) and of the COXIV subunit increased in Nrf2/WT trained animals. At the same time, Nrf2/KO animals experienced a reduction of NQO1. The study suggested that Nrf2 transcription encouraged by physical exercise is essential for protein stoichiometric adaptations, the enzymatic activity of ETC, and energy metabolism [67].

Wang et al. [67] reported that the stimulus of acute exercise promoted the signaling pathway of the redox effector factor 1 (Ref1) and Nrf2 in groups of male ICR/CD-1 mice. The main findings included the role that H_2_O_2_ mitochondrial content exerts on the elevation of OS and the activation of Ref1/Nrf2 signaling in a time-dependent manner (45, 90, 120, or 150 min), together with the expression of the antioxidant system: MnSOD and CuZnSOD activities; GSH content; the expression of the Ref1 and Nrf2 genes, and Ref1 and Nrf2 proteins in mouse skeletal muscle (gastrocnemius and quadriceps femoris). This suggested that acute exercise increases H_2_O_2_ and OS, enhancing the Ref1/Nrf2 pathway and antioxidant defense, basically GSH and MnSOD (mitochondrial isoform). Merry and Ristow [68] found that the deficiency of Nrf2 in mouse gastrocnemius muscle and muscle cells affects the production of the messenger RNA (mRNA) of Nrf1 and TFAM (mitochondrial biogenesis biomarkers), CAT, SOD1, and SOD2 in acute exercise. Additionally, after a five-week treadmill training protocol, mice deficient in Nrf2 (Nrf/KO) experienced an important decline in mitochondrial mass, SOD activity, and whole-body energy expenditure compared with Nrf2/WT. In isolated muscle-cell C2C12 myoblasts, acute exogenous exposure to H_2_O_2_ and diethylenetriamine/NO adduct (NO donor) increased TFAM. These results suggest that ROS and NO are able to regulate the Nrf2 pathway to stimulate mitochondrial biogenesis and the expression of antioxidant protection genes [68].

Mitochondrial ROS production controls a variety of physiological processes within the cell, regulating gene expression and signal transduction. The protein p^66Shc^ is a redox regulator of mitochondrial ROS, acting as H_2_O_2_ producer by sequestering electrons from ETC. It modulates H_2_O_2_ and proton leak and membrane oxidase activity, thus contributing to mitohormesis in mammalian cells [69,70]. Acute exercise appears to increase the expression of p^66Shc^ and transcription factor FOXO3a in correlation with H_2_O_2_ and in a time-dependent manner in gastrocnemius and quadriceps femoris of male ICR/CD-1 mice. Mitochondrial CAT was also affected in a time-dependent manner, but not SOD, which was not significantly modified [71]. FOXO3a is a FOXO family protein implicated in stress modulation and metabolism by interfering in SOD and CAT expression. FOXO3a is also related to the survival of organisms and lifespan extension under energy-nutrient restriction [70]. It is noteworthy that some evidence indicates that there is a more efficient cellular response to stress in trained compared with untrained individuals. Better skeletal-muscle oxidative capacity has been reported with lifelong endurance-exercise training after a single bout of cycling at 75% VO_2max_ in elderly trained individuals (aged 71.3 ± 3.4 years). Different indicators, such as PGC-1α, VEGF mRNA, and AMPK phosphorylation, were significantly higher in trained individuals. These results suggest an improved oxidative muscle capacity and a better ability to respond to acute endurance exercise [72]. It must be noted that single-bout exercise or regular exercise elicits the activation of numerous circuits closely related to mitohormetic adaptations.

### 3.3. Mitochondrial Uncoupling and Exercise

Mitochondrial uncoupling was initially correlated with mitochondrial dysfunction because it is the dissociation between the generation of membrane potential and the use for ATP synthesis. To a certain degree, uncoupling is necessary to explain diverse biological processes [73]. The lack of coupling could be explained by three possible situations: proton leak, electron leak, and electron slip. Proton leak basically depends on the composition of the inner mitochondrial membrane and can be modulated by a group of selective proteins: the uncoupling proteins (UCP). In addition, the electron leak process in ETC leads to the synthesis of O_2_^•−^ or of the hydroperoxyl radical (HO_2_^•^). Finally, electron slip is the transfer of electrons through the mitochondrial complexes without proton pumping [74,75]. 

In humans, UCP comprises a protein family constituted of five members (UCP-1–UCP-5). UCP-1, a transmembrane protein that catalyzes proton transport across the mitochondrial membrane, in turn leading to mitochondrial uncoupling, is found in the inner mitochondrial membrane [76]. UCP-1 displays an important role in thermogenesis in brown and beige adipose tissue and the elevation of mitochondrial ROS activates acute thermogenesis. Thermogenic ROS can modify cysteine thiols (cys253) to increase respiration in brown adipose tissue. The sulfenilation of cys253 by mitochondrial ROS supports UCP-1-dependent thermogenesis and the whole-body energy expenditure [77]. Exercise increases uncoupling and mitochondrial biogenesis in skeletal muscle, augmenting the mitochondrial uncoupling-driven thermogenesis and thus energy expenditure [73]. Acute exercise increases the expression of UCP-1 in brown adipose tissue of high fat diet-treated Swiss mice and ICR mice. The thermogenic effect is explained by the enhancement of leptin-induces hypothalamic ERK1/2 phosphorylation, which stimulates thermogenesis in brown adipose tissue [78].

UCP-2 was found to be activated, for instance, is a massive entry of free fatty acids (FFA) into mitochondria [79], similarly to UCP-3, which has been found to be elevated in situations of starvation [80]. The latter is expressed in rat and mouse skeletal muscle; it was demonstrated that the content of UCP-3 in the intermyofibrillar fraction is 1.3-fold higher compared with the subsarcolemmal mitochondrial fraction. In addition, under conditions of stress, in the presence of ROS, UCP-3 promotes the Nrf2 antioxidant response and diminishes superoxide generation by increasing the proton conductance of the internal mitochondrial membrane [81]. UCP-3 is expressed 200–700-fold lower than UCP-1 in brown adipose-tissue mitochondria from warm-adapted hamsters (24–84 µg of UCP-1/mg of mitochondrial protein) [82]. There are some contradictory results found by Nabben et al. [83] in models of acute lipotoxicity–fatty acid-induced oxidative inhibition and fatty acid-induced swelling, in which no evidence was found with regard to UPC-3 protection against OS, ROS regulation, or the protection of acute lipotoxicity in UCP-3 (−/−) mice, backcrossed for 10 generations on a C57Bl/6 background and isolated skeletal-muscle mitochondria.

UCP-3 is expressed in response to exercise and other factors, such as that fat and glucose metabolism modulate its expression during moderate exercise. A rapid biphasic alteration has been observed of UCP-3 gene transcription after 1 h of moderate exercise, and UPC-3 upregulation was sustained for 3 h during the recovery period [84]. In prolonged exercise, that is, 45, 90, 120, and 150 min of a single bout of exercise, UPC-3 expression is rapidly upregulated in rat skeletal muscle, probably due to the gradual increase of intracellular ROS. Due to UCP-3 upregulation during exercise, it is thought that the former potentiates OxPho because it alleviates the proton gradient across the inner mitochondrial membrane, thus reducing further ROS generation via the ETC. The continued physical activity caused a decline in energy-coupling efficiency observed by a rise in respiration rate as a consequence of proton leak and a decrease in the respiratory control index (RCI). In turn, it has been proposed that this is another way of how UCP-3 may act as an antioxidant to protect the mitochondria in muscle cells from exercise-induced OS [85]. In parallel, acute or severe hypobaric hypoxia appears to upregulate UCP-3 and may attenuate the cross-membrane potential (Deltapsi). Through endurance, exercise improves the mitochondrial OxPho by means of better ROS elimination and diminishes hypoxia-induced UPC-3 upregulation [86].

## 4. Mitochondrial Damage in Exercise Performance

The importance of mitochondrial function is currently being constantly mentioned in terms of maintaining optimal cell conditions. In agreement with hermetic theory, a certain level of stress is required to stimulate or modulate molecular signaling to enhance cell functioning and survival in order to withstand greater insults. Exercise-induced stress is manifested in different forms, including mechanical, metabolic, thermal, and OS, which activates molecular messengers serving as potential boosters of the signaling pathways that regulate adaptative responses to physical training [87]. The big question now appears to be on how to determine the amount of stress necessary to induce cellular adaptations and when the adaptive threshold is exceeded, and cellular damage starts. That is, at what moment are the cytoprotective defenses of the antioxidant system surpassed?

From the bioenergetic perspective, mitochondria comprise the cell core in every single reaction in which ATP is required; thus, a constant oxygen supply to fuel muscle-force production must be achieved. In turn, reducing the OxPho capacity will directly affect the athletic physical performance, contributing to fatigue and the previously mentioned OTS. Mitochondrial-reduced capacity can be the cause of restricted VO_2max_ and the impairment of performing high-intensity sports activities. Very limited studies have been conducted to elucidate the relation between mitochondrial damage indicators and the detriment of athletic performance. The study conducted by Kadaja et al. [88], in a six-week treadmill exercise program to determine changes in energy metabolism in rat myocardium, revealed that chronic exhaustive exercise induces the presence of OTS accompanied by muscle catabolism, body-weight loss, and decreased performance. 

It is noteworthy that maximal performance as reflected in physical working capacity was reported in week 3, but in week 6, this decreased remarkably: the animals were unable to maintain that training load for a longer period. Regarding heart–weight changes, a rise was observed to an increased heart-to-body weight ratio, indicating that overtraining induces anabolic responses in the myocardium. Microscopic analysis showed significant myocardium changes, including the disintegration of cardiomyocyte structure, the appearance of peroxisomes, and cellular swelling. Mitochondrial structures appeared to be intact, but had separated from the myofibrils, suggesting that the cytoskeleton surrounding the mitochondria may have disintegrated. Furthermore, the appearance of the peroxisomes, manifested as rounded, electron-dense dark bodies, could be associated with exercise-induced stress. In addition, a decrease was described in OxPhos by means of a reduction in cytochrome c and the impaired coupling of adenylate kinase (AK) to OxPhos. These experiments suggested that overtraining impairs functional coupling between mitochondrial AK2 and ANT protein and produces a drop in the overall capacity of ADP-dependent OxPhos. The study points out that an excessive work volume could result in OTS affecting the myocardium as well.

**Figure 2:**
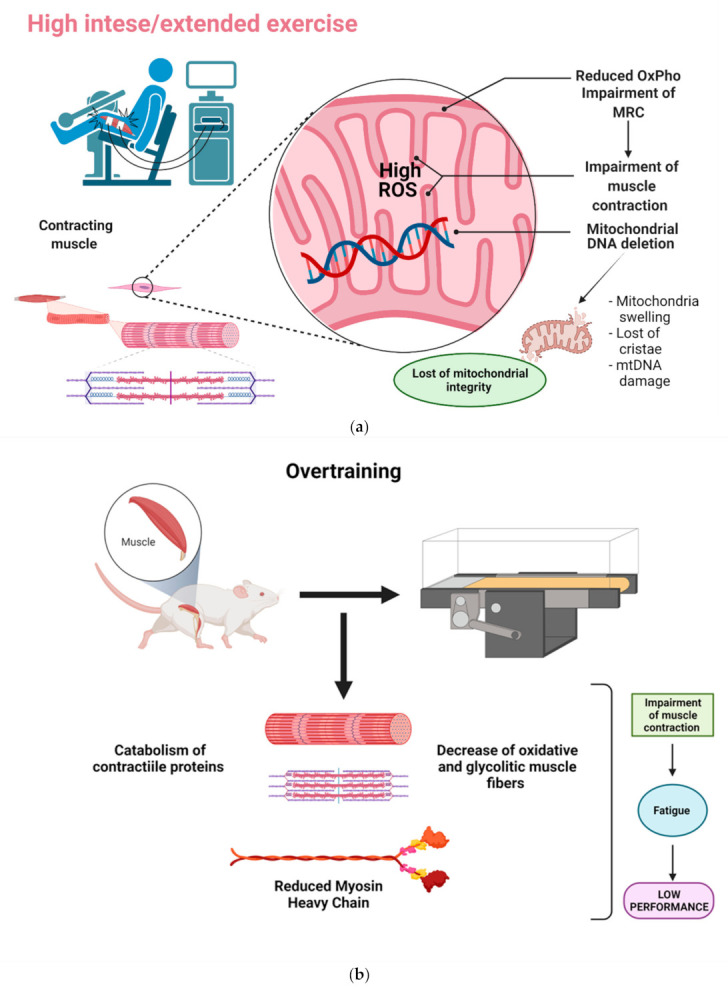
Impact of mitochondrial damage in exercise performance. (**a**) Graphical representation of mitochondrial reduced function and impairment of muscle contraction in intense or exhausting exercise. (**b**) Description of overtraining and the impact on muscle catabolism. ROS: reactive oxygen species; OxPho: oxidative phosphorylation; MRC: mitochondrial respiratory chain. Created with BioRednder.com (accessed on 24 October 2021).

The reduced ability to perform high-intensity or extended exercises in athletes with high fatigue ratings and OTS has been positively correlated [89]. Reduced mitochondrial respiration may perhaps contribute to the impairment of muscle contraction and, in turn, physical performance (Figure 2a). Vigorous exercise could also lead to mitochondrial DNA deletion and apoptosis [90] and additionally to the mitochondrial structural changes observed as well as the swelling and loss of cristae after a single bout of extraneous exercise [91]. Exhaustive swimming exercise in rats during 10 weeks gave rise to muscle and myocardium damage, reported as the loss of mitochondrial and sarcoplasmic- or endoplasmic-reticulum integrity and the augmented lipid peroxidation of tissue homogenates in exhausted trained and untrained rats [92]. In addition, overtraining might impact muscle metabolism balance by promoting catabolism on contractile protein synthesis (Figure 2b). The overtraining protocol in rats led to a marked quantitative decrease of the slow-twitch oxidative fibers of the soleus muscle and of the fast-twitch glycolytic fibers of plantaris and extensor digitorum longus muscles, body-weight loss, a reduced rate of myosin heavy chain (MHC) synthesis, and an increase of excreted urinary 3-MeHis [93]. On the other hand, after 11 weeks of severe endurance training, male Wistar rats experienced a reduction in performance of 38% in the nonfunctional overreaching group (NFOR) compared with the functional overreaching group (FOR). In this study, remarkably lower citrate-synthase activity was observed, as well as the lower activity of mitochondrial complex IV in the red gastrocnemius muscle of the NFOR group. Cardiomyocyte apoptosis was also described in endurance overtraining as higher in the NFOR than in the FOR group [94].

After an eight-week training protocol of acute exhaustive exercise in rats conducted by Fang et al. [95], mitochondrial ROS production significantly increased, and the activity of respiratory complex V and ATP content in samples of skeletal muscle and brain was reduced. Acute exhaustive training decreased the expression of prohibitin-1 (PHB1) protein in mitochondria, OxPho was reduced, and the energy metabolism in general mitochondrial function was affected. The PHB comprises a phosphoprotein complex required for mitochondrial homeostasis and survival identified in human T-cells; PHB participates in cell activation, differentiation, function, and viability [96]. Different evidence reveals that mitochondria as the main supplier of ROS throughout exercise might be a central target of OS damage and the consequent undergoing of structural and functional disruptions. The analysis of mitochondrial fractions has demonstrated a proportional relation of the amount of damaged mitochondria with the extension of training bouts [97].

Taken together, this information clearly outlines that these mitochondrial responses to exercise could take two different paths: (1) mitochondrial adaptations in relation to low/moderate exercise, or (2) mitochondrial damage in response to excessive exercise workloads. In this manner, mitochondrial responses will depend to a great degree on the duration, intensity, and the frequency of exercise bouts. Low/moderate exercise promotes mitochondrial biogenesis and with no noticeable mitochondrial impairments such as swelling or disruption of cristae [98,99]. In contrast, exhaustive exercise is associated with cell damage, and with morphological alterations observed as flocculent mitochondria, swelling, incomplete myofibrils, and irregular sarcomeres [100]. Mitochondrial integrity must be a targeted issue in the search for optimal performance beyond adaptations to exercise.

## 5. Nutrition Insights into Mitochondrial Protection

### 5.1. General View of Antioxidants, Oxidative Stress, and Exercise Performance

For many years, there has been a constant debate on whether the use of antioxidants truly benefits exercise performance/recovery or impairs biological adaptations to heavy training [101,102,103]. Current available data point out that antioxidant requirements should be covered by a balanced and individualized diet, with the individual consuming the recommended dietary allowance (RDA) doses. Nonetheless, under special circumstances such as high-intensity or prolonged exhaustive sports, antioxidant supplementation can be an optimal way to prevent or reduce the consequences of damage, such as unchaining the previously mentioned fatigue and/or OTS [104,105].

Frequently, antioxidant supplementation is addressed in terms of improving athletic performance, but the evidence is contradictory. It should take into consideration that reducing OS is not necessarily associated with the enhancement of exercise performance. In a double-blind controlled assay, supplementation of vitamin C (500 mg/d) and E (400 IU/d) for 15 days in football players did not modify muscle damage or muscle soreness after acute exercise-induced OS. The reported findings did not exhibit any ergogenic effect; nevertheless, OS was attenuated [106]. Similarly, supplementation with vitamin C and E in improved indices of OS was associated with repetitive loading exercise by increasing the activity of the antioxidant enzyme MnSOD in young animals. CuZnSOD and CAT activity also increased in young and aged animals and improved the positive work output of muscles in aged rodents. These data suggest that more regulatory factors of OS are implicated, such as age and exercise type, which indirectly influence physical performance [107]. 

On the other hand, four-week antioxidant supplementation with vitamin C (2 × 500 mg/day) and E (400 IU/day) did not exert any effect on skeletal-muscle oxidative OS indicators or in the expression of mitochondrial biogenesis genes (PGC-1α, TFAM, or PGC-related factor). No changes in VO_2max_, citrate-synthase activity, and mRNA cytochrome oxidase subunit IV (COX IV) in skeletal muscle were observed. In contrast, antioxidant supplementation attenuated skeletal-muscle adaptation biomarkers in SOD activity and the protein abundance of TFAM and SOD 2 [108]. Earlier, it was informed that vitamin C and E supplementation blunts adaptations to endurance training in human trials [109].

Polyphenols are a huge group of molecules that have been largely investigated for their multiple health benefits [110], but their tentative impact on OS and exercise performance remains unclear [111]. Grape polyphenols from beverages, extracts, or supplements have exhibited very good results for the reduction of damage in the intense exercise of diverse sports activities [112]. Lately, some other phytochemicals appear to be potential compounds that could exert an impact on exercise performance and the lowering of OS in some way by their attributed antioxidant activity. For instance, curcumin at doses of 500 mg/day for 8 weeks significantly improved total antioxidant capacity (TAC), malonaldehyde, serum C-reactive protein (CRP), and VO_2max_ in physically active women [113]. 

Our research group found that silymarin, the flavonolignan extract from Milk thistle, has been demonstrated to increase physical performance and to promote muscle hypertrophy and recovery at doses of 100 mg/kg after eight weeks of regular exercise in a rodent model [114]. Aside, the pretreatment with sulforaphane (SFN) from broccoli in 13-week-age Nrf2+/+ and Nrf2−/− male mice upregulated Nrf2 signaling and downstream genes, improved the endurance capacity associated with a lower OS marker, and provided protection against muscle fatigue during exhaustive exercise. SFN may enhance exercise performance via upregulation of the Nrf2 pathway after an exhaustive treadmill test (progressive-continuous all-out [115]. To date, very few investigations have been conducted on the benefits of SFN in exercise; however, SFN is thought to be a promising molecule on providing an ergogenic effect on exercise performance because preceding literature supports SFN effectiveness in prompting the Nrf2/Keap1/ARE signaling pathway and in suppressing NF-κΒ signaling. Therefore, SFN has great potential for offering protection against OS, inflammation, and fatigue [116]. Of note, silymarin and curcumin have also been reported to be effective bioactivators of Nrf2/Keap1/ARE in a variety of in-vitro and in-vivo studies [117,118,119].

### 5.2. Mitochondria as Targets for Protection from Oxidative Damage

At present, substantial evidence indicates that DNA damage appears immediately after acute aerobic exercise but that the potential DNA renewal begins after day 3. The relationship concerning what supports the explanation of exercise-induced DNA damage must be understood beyond the hormesis theory, which refers to ROS/RNS as key actors of genomic damage surrounding the breaking of the balance between physical inactivity and overtraining. A multi-dimensional overview has been recently proposed to build a better approach for the complex relationship between DNA integrity and exercise [120]. According to the authors, the multi-dimensional model includes the whole perception of multiple factors regarding the extension of DNA oxidative damage that the exercise-induced. Eight main factors are listed as follows: (1) type of ROS/RNS, from the least to the most reactive; (2) frequency of attacks or episodes of exposure to ROS/RNS; (3) type and extension of DNA damage (single/double-strand breaks); (4) magnitude of DNA oxidation by ROS/RNS; (5) exercise intensity/distance; (6) frequency of training bouts; (7) DNA repair enzymes, and (8) degree of individualization, which comprises further issues, such as age, sex, training level, and the nutrition quantification method [120]. 

This entire view would be theoretically applicable to the analysis of mtDNA damage caused by exercise, considering that there are certain elements that could render mtDNA more susceptible to damage in comparison with nuclear DNA, such as the accumulation of transition metal ions, the secondary subproducts of DNA, lipid oxidation, and finally, the absence of histones and chromatin organization. More studies are required to determine the mechanism implicated in mtDNA damage as a result of exhaustive/intensive exercise. 

An understanding of the importance of mitochondrial dynamics in bioenergetics, signaling, and adaptation to exercise, mitochondrial integrity must be an elemental issue in developing target antioxidants. Mitochondrial dysfunction is being associated with several common pathologies, such as metabolic and cardiovascular diseases, ischemia–reperfusion injury, and neurodegeneration; thus, the mitochondrial features have been largely studied as a crucial drug target to treat the previously mentioned disorders [121]. Several therapeutic approaches are emerging in which some compounds have been designed to provide the target of mitochondrial protection.

Although the effectiveness of conventional antioxidant supplementation and exercise-induced OS has been well-described [122,123,124], there is an absence of evidence regarding mitochondrial-targeted antioxidants in exercise. An example of this is the following double-blind, randomized trial. Twelve healthy male participants (age = 28 ± 10 years, height = 177 ± 12 cm, and body mass = 81 ± 15 kg) were assigned to receive either a daily supplement of 1000 mg of α-lipoic acid (2 × 500-mg tablets) for 14 days or to receive no supplement. The participants performed 100 isolated, continuous, maximal knee extensions (minimal force = 200 N, speed of contraction = 60°·s^−1^). The analysis of blood and muscle biopsy tissue samples revealed that the exercise promoted mtDNA damage by significantly increasing the 8-hydroxy-2-deoxyguanosine (8-OHdG) concentration; in parallel, total antioxidant capacity was significantly reduced in both groups. An augmented total antioxidant capacity was found in blood samples; the comet essay and 8-OHdG analysis confirmed DNA protection and reduced lipid peroxidation and hydroperoxides in the supplemented group. Both groups experienced protein oxidation indistinctly. The findings suggested that short-term α-lipoic acid supplementation may protect nuclear DNA and lipids, but not mtDNA, in high-intensity, isolated, single-leg exercise. The tentative protective role of α-lipoic acid in blood oxidative markers is perhaps independent of muscle OS indicators and shelters DNA integrity selectively. These results may be associated with a different antioxidant baseline ability within the cell compartment [125].

### 5.3. Target Mitochondrial Antioxidants in Exercise-Induced Oxidative Damage

**Figure 3:**
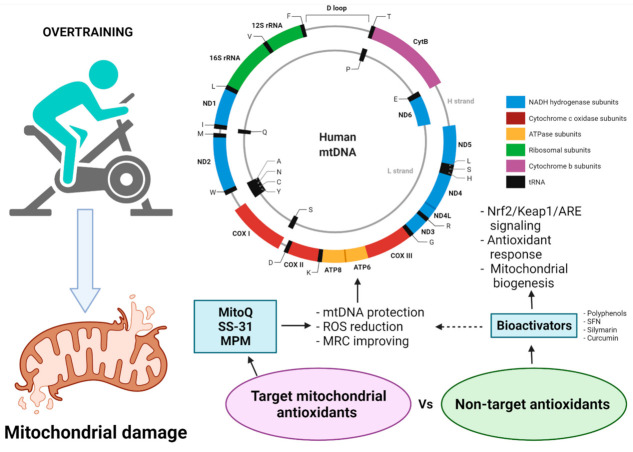
Efficacy of non-target versus target mitochondrial antioxidants supplementation and their effect on exercise performance. MCR: mitochondrial respiratory chain; MPM: micropeptide in mitochondria; SFN: sulforaphane. Created with BioRender.com (accessed on 24 October 2021).

In this respect, several factors must be considered, such the inherent mitochondrial biological nature and the chemical nature, such as the molecular dynamics of the bioactive compounds when they are intended to be tested as possible mitochondrial target protectors. Mitochondria possess morphological and physiological features that render them difficult to reach (Figure 3). In addition, bioactive compounds should meet different aspects in order to ensure arrival at the target-cell compartment, and these aspects are herein mentioned as follows: (1) the issue of bioavailability; (2) transportation to the target organelle, and (3) the ability to cross mitochondrial barriers in order to be able to deliver it to the site-of-action. These are the possible limitations that general antioxidants may frequently face in the attempt to exert an antioxidant effect within the mitochondria. Therefore, different current forms of target antioxidants have been designed, such as lipophilic cations, liposome enclosure, and target peptides. Examples of these target antioxidants employed in clinical trials are MitoQ [126,127,128], MitoE [129,130], tiron [131], and MitoC [132]. For an integrative overview of target mitochondrial antioxidants in exercise-induced DNA damage, see the recommended review [133].

The difference between mitochondrial-target antioxidants and untargeted antioxidants is the potential to remove ROS/RNS from the production source due to the ability to cross the mitochondrial phospholipid bilayer [134]. MitoQ is an orally available mitochondrial-target coenzyme Q10 that contains the antioxidant quinone moiety covalently attached to a lipophilic triphenylphosphonium cation. MitoQ has been applied in a variety of in-vivo studies in rodent models and in diverse human trials [135]. MitoQ has the ability to mimic the antioxidant activity of coenzyme Q (CoQ) and simultaneously increase to supraphysiological levels in a mitochondrial-membrane potential-dependent manner to approximately 100–1000 times greater than non-target derivatives. MitoQ is positively charged, possesses a large molecular weight and is highly lipophilic and low water-soluble. These properties allow MitoQ to pass easily through biological membranes, promoting its mitochondrial accumulation to several hundred-fold, encouraged by the mitochondrial membrane potential [136,137,138]. MitoQ can be accumulated in the inner mitochondrial membrane at the matrix-facing side, where it is reduced by complex II to ubiquinol (MitoQH_2_) [139]. These localization abilities permit MitoQ to donate hydrogen atoms to oxygen, or even α-tocopherol radicals; thus, it can be effective against lipid peroxidation and peroxynitrite, preventing or attenuating oxidative damage in mitochondrial lipids, proteins, or mtDNA [140]. Consequently, it represents a potential way to deliver precise cytoprotection against exercise-induced oxidative damage.

The study of MitoQ in the context of the exercise was first explored by Shill et al. in 2016 [141] and, after three weeks of MitQ supplementation (10 mg/day), the authors demonstrated that skeletal-muscle and whole-body adaptation to endurance training was not affected by MitoQ in young, healthy men by the absence of a significant difference in VO_2max_ oxidative capacity and circulating angiogenic cells. Recently, the novel work of Williamson et al. [142] demonstrated, in a double-blind placebo controlled study, that in a high-intensity intermittent exercise (HIIE), randomized, placebo-controlled design, 24 healthy male participants divided into two groups (placebo; *n* = 12, MitoQ; *n* = 12) were subjected to an exercise trial of 4 × 4-min bouts of cycling at 90–95% of maximal heart rate. The participants received an acute treatment of 20 mg-MitoQ or placebo 1-h pre-exercise and a chronic 21-day phase of supplementation. The results indicated that HIIE induced mtDNA damage in lymphocytes and in human-muscle tissue that was accompanied by modifications in lipid hydroperoxides, α-tocopherol, and ascorbyl free radicals. The MitoQ acute treatment was not sufficiently efficient to protect against mtDNA damage, possibly due to the low bioavailability. Notwithstanding, chronic MitoQ supplementation protected nuclear and mtDNA damage; the likely mechanisms implied that MitoQ helps to metabolize ROS, and that it may also safeguard complexes I and IV (cytochrome c complexes) in the ETC from oxidative damage. 

MitoQ may have also interacted with proton-motivated forces (uptake reduces proton-motivated force) and favored the coenzyme Q-pool redox state, which possibly affected complex III-mediated superoxide production. In addition, MitoQ could be helping in extramitochondrial activities and in potential participation in metabolizing cytosolic H_2_O_2_. This research offers a new understanding with regard to mechanistic insights into mitochondrial redox dynamics and targeted supplementation to safeguard nuclear and mitochondrial genomes following HIIE. MitoQ is the best example of how to target antioxidants elicit benefits in protection against oxidative damage due to heavy training; however, the ability of MitoQ would be opposite to this if mitochondrial concentrations were increased to 0.5–1 µM vs. 2 µM on H_2_O_2_ production. It is noteworthy that when the balance between antioxidants and prooxidants is broken, we again have the same concern; that is, when cellular situations of susceptibility, such as unstable lipid membranes, quinone-derived compounds might propagate lipid peroxidation within the mitochondrial membrane [143,144]. This is possible because mitochondria are highly predominant in lipids susceptible to peroxidation, such as anionic cardiolipin, phosphatidylethanolamine, and phosphatidylcholine [145].

Park et al. [146] reported that the acute oral intake of MitoQ (80 mg) improved maximal walking capacity and postponed the onset of limping in individuals with peripheral artery disease. The authors also observed increasing vascular endothelial function and SOD. Although this research was not conducted exclusively in the context of high-intense or extended exercise, it was, to the authors’ knowledge, the first study of mixed exercise-targeted antioxidant supplementation with pathological variables. This highlights that several studies indicating the prophylactic effect attributed to MitoQ are related to the upregulation of the Nrf2/Keap1/ARE signaling pathway and the target antioxidant genes HO-1, NQO, GPx, GR, Trx, CAT, and γ-GCLC [126,128,147], which could also be involved in the complex network of cytoprotection in conjunction with the target mitochondrial antioxidant activity. It is also important to bear in mind the role that Nrf2 signaling plays in mitochondrial biogenesis and adaptation, together with other transcriptional factors, such as PGC-1α, NF-κΒ, PPARα, EERα, TFAM, among others, that we have already discussed. Therefore, it would be very limiting to only address the benefits of MitoQ in terms of a single mode-of-action.

Other interesting molecules appear attractive with respect to the Szeto–Schiller (SS) peptides (SS-19, SS-02, SS-31, and SS-20), which have been analyzed in some trials: these peptides also provide an antioxidant effect attributed to their tyrosine or dimethyl tyrosine residue, which allows them to scavenge ROS easily. These molecules are able to accumulate in the inner mitochondrial membrane at a concentration of 1000–5000-fold by means of hydrophilic and hydrostatic interactions; thus, they are able to cross cellular and mitochondrial membranes because they also possess the alternating aromatic–cationic amino-acid motif [148]. It appears that SS-31 has been mainly efficient according to the data because of its ability to attach to and protect against cardiolipin peroxidation by shifting cytochrome c peroxidase activity [149,150]. In addition, it can scavenge peroxynitrite and hydroxyl radicals due to the addition of dimethyl tyrosine [151]. In 14 days of inactivity-induced muscle atrophy, SS-31 supplementation mitigated mitochondrial ROS, protease activation, and muscle atrophy; to a great extent, skeletal-muscle atrophy is linked to modifications in cytosolic free Ca^2+^, inducing the activation of caspase-3 and calpain [152].

Peptides also comprise an innovative topic as a goal in mitochondrial protection; micropeptides are small molecules of <100 amino acids with specific functions [153]. Most recently, a novel muscle-enriched micropeptide was identified that was localized to mitochondria and that is denominated a micropeptide in mitochondria (MPM), identified in a ClC2 myoblast. The silencing *MPM* gene (MPM−/−) in mice permitted the recognition of its implication in myogenic differentiation and in muscle-fiber growth when the expression of *Pax7*, *MyoD*, and *MyoG* expression was impaired in MPM−/−. Furthermore, MPM−/− mice exhibited smaller skeletal-muscle fibers and worse muscle performance, manifested by a reduction in the maximal grip force of limbs, the latency to fall off the rotarod, and the exhausting swimming time. These effects were due to that *MPM* is involved in mitochondrial respiratory activity and mitochondrial biogenesis simultaneously, thus becoming a suitable target for therapy for muscle dystrophy or perhaps as a therapy for other muscle disorders [154]. 

Finally, while bioactive compounds from foods or plants, probably similar to those that we have mentioned before, including polyphenols, SFN, curcumin, and silymarin, do not fit into the categorization of target antioxidants, have the potential for very profound exploration in an attempt to elucidate their possible effects on mitochondrial protection and possible interactions within cell signaling, such as activation of the Nrf2 pathway [155,156].

## 6. Conclusions and Perspectives

The oxidative stress induced by an imbalance between ROS/RNS production and antioxidant systems has been well described in many pathological conditions, such as cancer, diabetes, cardiovascular diseases, immunological, neurodegenerative disorders, and aging. Moreover, as a consequence of increased oxygen consumption and energy demands through physical exercise, more ROS/RNS are produced, unchaining a series of cellular events related to the antioxidant response, molecular signaling linked with adaptation, and other biological responses. It is also known that high-intensity or exhaustive exercise might surpass the EnAS, raising oxidative stress, inflammation, and skeletal-muscle damage that, under chronic exposure, may eventually lead to fatigue and OTS, affecting performance.

Despite the vast literature with respect to exercise-induced muscle damage, few investigations have addressed mitochondrial damage and nutrition-specific strategies. Supplementation with antioxidants received particular attention as an essential tool for preventing or attenuating the damage induced, thus probably exerting a positive impact on sports performance [157]. However, evidence is highly contradictory concerning whether antioxidants truly benefit performance due to their protection or to their impairing adaptations to exercise. Mitochondria comprise one of the major ROS/RNS producers; therefore, they are highly susceptible to being attacked, and EnAS would not be sufficient to counteract mitochondrial damage, leading to a detriment in the performance of exercise and recovery.

Building on the limited body of literature, this review provides new insights into the role of exercise-induced redox perturbations in mitochondria, along with a new perspective on mitochondrial-targeted supplementation, which represents a novel area to explore. At the same time, there are a large number of bioactive compounds that could also offer protection by triggering the signaling involved in cytoprotection, mitochondrial dynamics, mitohormesis and biogenesis. It is noteworthy that the cytoprotector response is the result of constant crosstalk between EnAS and ExAS, and efficacy is notably based on arriving at a systemic equilibrium and an attempt to preserve this balance to the degree possible.

In conclusion, future research should take into consideration that mitochondrial oxidative damage might intervene in cellular adaptation, but that the supplementation of antioxidants might influence exercise performance. To date, it continues to be difficult to reach a conclusion in terms of antioxidant supplementation in athletes. Nonetheless, the use of antioxidants for mitochondrial protection against exercise-induced oxidative damage remains an innovative field to investigate from different angles.

## Data Availability

Not applicable.
